# Quantitative RNA-seq analysis of the *Campylobacter jejuni* transcriptome

**DOI:** 10.1099/mic.0.050278-0

**Published:** 2011-10

**Authors:** Roy R. Chaudhuri, Lu Yu, Alpa Kanji, Timothy T. Perkins, Paul P. Gardner, Jyoti Choudhary, Duncan J. Maskell, Andrew J. Grant

**Affiliations:** 1 Department of Veterinary Medicine, University of Cambridge, Madingley Road, Cambridge CB3 0ES, UK; 2 Wellcome Trust Sanger Institute, Wellcome Trust Genome Campus, Hinxton, Cambridge CB10 1SA, UK

## Abstract

*Campylobacter jejuni* is the most common bacterial cause of foodborne disease in the developed world. Its general physiology and biochemistry, as well as the mechanisms enabling it to colonize and cause disease in various hosts, are not well understood, and new approaches are required to understand its basic biology. High-throughput sequencing technologies provide unprecedented opportunities for functional genomic research. Recent studies have shown that direct Illumina sequencing of cDNA (RNA-seq) is a useful technique for the quantitative and qualitative examination of transcriptomes. In this study we report RNA-seq analyses of the transcriptomes of *C. jejuni* (NCTC11168) and its *rpoN* mutant. This has allowed the identification of hitherto unknown transcriptional units, and further defines the regulon that is dependent on *rpoN* for expression. The analysis of the NCTC11168 transcriptome was supplemented by additional proteomic analysis using liquid chromatography-MS. The transcriptomic and proteomic datasets represent an important resource for the *Campylobacter* research community.

## Introduction

*Campylobacter jejuni* is the most common bacterial cause of foodborne disease in the developed world, with an estimated one in every 100 individuals in the UK developing a *Campylobacter*-related illness each year, resulting in an economic burden of up to £500 million per annum (Humphrey *et al.*, 2007). Although most cases are self-limiting, *C. jejuni* has been associated with severe post-infection complications, including polyneuropathies such as Guillain–Barré syndrome (Goodyear *et al.*, 1999). The bacterium is a common gut commensal of animals destined for human consumption, and faecal contamination of meat during processing is a recognized route of transmission to humans (Corry & Atabay, 2001; Herman *et al.*, 2003). *C. jejuni* (NCTC11168) was the first food-borne pathogen to be sequenced (Parkhill *et al.*, 2000) due to its importance as a pathogen and the fact that it has a relatively small genome (1.6 Mb). Many other *Campylobacter* species have been sequenced, and these genome sequences have facilitated many functional genomics-based approaches to study the roles of individual genes and proteins in colonization, environmental survival and virulence.

An important step in the control of bacterial gene expression is the initiation of transcription, which is dependent on the DNA-dependent RNA polymerase core enzyme binding a sigma factor (Polyakov *et al.*, 1995). Unlike *Escherichia coli*, which has seven sigma factors, the genome sequence of *C. jejuni* NCTC11168 revealed the presence of only three (Parkhill *et al.*, 2000): RpoD (σ^70^), responsible for the transcription of most genes (Petersen *et al.*, 2003), and RpoN (σ^54^) and RpoF/FliA (σ^28^), which control the expression of genes required for flagellum biosynthesis and chemotaxis (Hendrixson *et al.*, 2001; Jagannathan *et al.*, 2001; Wassenaar *et al.*, 1994). The lack of sigma factors in *C. jejuni* suggests that alternative regulatory mechanisms must exist and/or that the two alternative sigma factors play wider regulatory roles than those characterized in other species. Although σ^54^ is known to be involved in the transcription of flagellar genes including those encoding the hook, basal body and minor flagellin (Carrillo *et al.*, 2004; Hendrixson & DiRita, 2003; Wösten *et al.*, 2004), the complete RpoN regulon in *C. jejuni* has not been fully defined.

*C. jejuni* remains a poorly understood pathogen and many of the paradigms that have been established with ‘model’ bacterial species such as *E. coli* and *Salmonella enterica* do not apply to it. Consequently, new approaches are required to understand this pathogen. Recently, several eukaryotes (Cloonan *et al.*, 2008; Mortazavi *et al.*, 2008; Wilhelm *et al.*, 2008) and prokaryotes (Perkins *et al.*, 2009; Yoder-Himes *et al.*, 2009) have been profiled at the transcriptome level using direct high-throughput Illumina sequencing of cDNA. This technique offers the ability to survey the entire transcriptome of an organism in a high-throughput and quantitative manner (Marioni *et al.*, 2008; Wang *et al.*, 2009). Since a large number of sequencing reads can be readily obtained, the method is sensitive and offers a large dynamic range. Thus RNA-seq can be used to detect and quantify RNA expressed at very low levels, in contrast with what can be found using DNA microarrays (Nagalakshmi *et al.*, 2008).

In this study we have used Illumina high-throughput DNA sequencing to study mRNA expression levels (i.e. the transcriptome) of *C. jejuni* NCTC11168, and have compared the transcriptome data with protein expression data. In addition we have used RNA-seq to map and quantify the transcriptome of an *rpoN* mutant. We have compared RNA-seq with existing microarray technologies for their abilities to identify differentially expressed genes, and we have identified putative small non-coding RNAs (ncRNAs) that would not have been apparent using microarray technology.

## Methods

### 

#### Bacteria.

*E. coli* strain DH5α was grown at 37 °C on Luria–Bertani (LB) agar or in LB broth. Preparation of electrocompetent *E. coli* cells and transformations were performed as described previously (Dower *et al.*, 1988). *C. jejuni* was routinely cultured on Mueller–Hinton (MH) agar (Oxoid) supplemented with 5 % defibrinated horse blood (hereafter referred to as MH blood agar plates) at 42 °C under microaerobic conditions (5 % O_2_, 5 % CO_2_, 5 % H_2_, 85 % N_2_) in a MACS VA500 variable atmosphere workstation (Don Whitley Scientific). Liquid cultures of *C. jejuni* were grown in brain heart infusion (BHI) broth (Oxoid) at 42 °C under microaerobic conditions with agitation at 150 r.p.m. Where necessary for selection, media were supplemented with chloramphenicol (10 µg ml^−1^), ampicillin (100 µg ml^−1^) or trimethoprim (10 µg ml^−1^).

#### Generation of *C. jejuni* NCTC11168 *rpoN* : : *cat* mutant.

The *C. jejuni rpoN* mutant was generated by insertional inactivation of the gene using a chloramphenicol acetyltransferase (*cat*) cassette. Standard methods were used for molecular cloning (Sambrook & Russell, 2001). Chromosomal and plasmid DNA purifications were performed using commercial kits following the manufacturers’ instructions (Qiagen). Routine DNA modifications including restriction endonuclease digestion of DNA, modifications of DNA and ligations were carried out according to the manufacturers, instructions (Promega, Invitrogen, Roche, New England Bioloabs). PCR primers were designed using Primer3 (http://frodo.wi.mit.edu/primer3/) and purchased from Sigma. All PCRs were performed in 50 µl reaction volumes in 0.2 ml Eppendorf tubes in an Applied Biosystems GeneAmp PCR Systems 9700 (Applied Biosystems). Reactions contained 200 µM dNTPs, 2 mM Mg^2+^, 0.01 vols Proof Start DNA polymerase (Qiagen; 2.5 U µl^−1^), 0.1 vols polymerase buffer (10×), 1 µM forward and reverse primers, and template DNA (~50 ng plasmid DNA or ~100 ng chromosomal DNA). Typical thermal cycler conditions were 94 °C for 4 min, 30 cycles of 94 °C for 1 min, 55 °C for 1 min and 72 °C for 1 min, followed by a final extension of 72 °C for 7 min. DNA and RNA concentration and purity were measured using a Nanodrop ND-1000 spectrophotometer. The *rpoN* gene was amplified by PCR from *C. jejuni* NCTC11168 chromosomal DNA using primers ajg285 (5′-GGGGGGATCCATGTTAAAGCAAAAAATCACC-3′) and ajg286 (5′-GGGGGGATCCTTATCCTTCAAGTTCATATAA-3′); the resulting fragment was cloned as a *Bam*HI fragment into *Bam*HI-digested pUC19 to generate the plasmid pAK1. To facilitate subsequent selections a chloramphenicol resistance gene with its promoter was amplified by PCR from pEnterprise2 (Hendrixson *et al.*, 2001) using primers ajg289 (5′-GGATCCCTTAAGCTCGGCGGTGTTCCTTTCCAA-3′) and ajg290 (5′-GGATCCCTTAAGCGCTTTAGTTCCTAAAGGG-3′) and inserted as an *Afl*II fragment into *Afl*II-digested pAK1 (*rpoN* contained a unique *Afl*II restriction site), generating pAK2. pAK2, with a *cat* cassette in the forward orientation relative to *rpoN*, was introduced into *C. jejuni* by natural transformation using a plate biphasic method adapted from Van Vliet *et al.* (1998). The structure of a representative isolate, with a chromosomally located *rpoN* : : *cat* insertion, was confirmed by PCR and Southern hybridization (data not shown).

#### Motility assays.

Motility assays were performed essentially as described by Silverman & Simon (1973). Briefly, a platinum wire was dipped into a single colony and used to stab MH motility medium containing 0.4 % agar. Motility was assessed by measuring colony diameter after incubation at 42 °C for 16 h.

#### RNA preparation for RNA-seq.

*C. jejuni* strains were cultured for 48 h on MH blood agar plates with antibiotics added as appropriate. Bacterial lawns were harvested in 1 ml BHI broth, 50 µl of which was inoculated into 10 ml BHI broth in a 50 ml Falcon centrifuge tube with a loosened cap, and grown for 16 h at 42 °C under microaerobic conditions with agitation at 150 r.p.m. to generate a starter culture. The OD_600_ of starter cultures was measured by using a 6305 UV/Visible Spectrophotometer (Jenway). Starter cultures were diluted appropriately in BHI broth and used to inoculate 10 ml BHI broth containing 10 µg trimethoprim ml^−1^ in a 50 ml falcon tube (previously equilibrated at 42 °C in microaerobic conditions for 16 h) to a calculated OD_600_ of 0.00002. Cultures were grown at 42 °C under microaerobic conditions with shaking at 150 r.p.m. for 24 h. Growth was monitored by recording OD_600_ and by determining viable c.f.u. ml^−1^ by serial 10-fold dilution of cultures in BHI broth and plating onto MH agar. Plates were incubated microaerobically at 42 °C for 48 h before colonies were counted. *C. jejuni* cultures were fixed with 2 vols RNA protect bacteria (Qiagen) and harvested. RNA was isolated from the pellet using the SV RNA isolation kit (Promega) according to the manufacturer’s instructions. 23S and 16S rRNA was depleted using a MicrobExpress kit (Ambion). Genomic DNA was removed with two digestions, using amplification-grade DNase I (Invitrogen), to below levels detectable by PCR. RNA was reverse-transcribed using random primers (Invitrogen) and Superscript III (Invitrogen) at 45 °C for 3 h and denatured at 70 °C for 15 min. *leuB* (using primers QJAW084 5′-GCAAGTATAGATGCTTATGGAGTG-3′ and QJAW085 5′-CTCTTTCAGGTCTTTGATCTATGG-3′), *aroA* (QJAW092 5′-GCTTTGGCTAAGGGTAAATCTAGT-3′ and QJAW093 5′-ATCAAGTTCTCTAGCTTCAACACC-3′), *lpxD* (QJAW104 5′-GGAGCTTATATAGGCGATAATGTC-3′ and QJAW105 5′-CAAAACCGTCACTTCCTATTACAC-3′) and *guaB* (QJAW108 5′-GGGTGTTGATGTTGTTGTGC-3′ and QJAW109 5′-GGCGATATTTCCTGCGATAA-3′) were used as targets for a PCR as a positive control for reverse transcription.

#### Library construction and sequencing.

Library construction and sequencing were carried out as described previously (Perkins *et al.*, 2009).

#### Read mapping and visualization.

Reads were mapped to the *C. jejuni* NCTC11168 genome sequence (GenBank accession no. AL111168) using Novoalign version 2.05 (Novocraft Technologies; http://www.novocraft.com) with default settings. Reads that mapped to more than one position in the genome were randomly distributed amongst the matching regions. Mapped reads were visualized using the version of BamView (Carver *et al.*, 2010) integrated into Artemis (Rutherford *et al.*, 2000). All subsequent analyses were performed using R version 2.8.0.

#### Quantification of transcript levels.

To estimate the level of transcription for each gene, the number of reads that mapped within each annotated coding sequence (CDS) was determined. To account for the possibility of unannotated ncRNA genes, the number of reads that mapped within each intergenic region of >150 bp was also determined. Reads that mapped within 50 bp of the adjacent genes were discarded to prevent the presence of 5′ and 3′ untranslated regions of adjacent transcripts from influencing the read count for the intergenic region. To enable comparison of expression levels, between both different RNA-seq experiments and different genes within the same experiment, it is necessary to normalize the read counts. The number of reads per kb of transcript per million mapped reads (RPKM) has been proposed as a useful metric that normalizes for variation in transcript length and sequence yield (Mortazavi *et al.*, 2008). RPKM values for each gene and intergenic region were calculated for the data from each Illumina lane, and mean RPKM values were determined for the wild-type and *rpoN* mutant samples. As RPKM values are log-normally distributed, it is convenient to express them as log_2_(RPKM+1). The complete dataset from this study has been deposited in the ArrayExpress database (http://www.ebi.ac.uk/arrayexpress) with the accession no. E-MTAB-706.

#### Analysis of differential expression.

Differential expression was assessed using DESeq (Anders & Huber, 2010). The total read count was determined for each gene and intergenic region by combining data from the two technical replicate sequencing runs. Read counts for the wild-type and the *rpoN* mutant were compared to determine the log_2_-fold change in abundance of each transcript. DESeq allows a *P*-value to be determined in the absence of any available biological replicates, by treating the two conditions as replicates, under the assumption that only a small proportion of transcripts is differentially expressed. *P*-values were calculated under this assumption, and adjusted for multiple testing using the false discovery rate controlling procedure (Benjamini & Hochberg, 1995).

#### Microarray analysis.

Raw data from an earlier *rpoN* mutant microarray study (Kamal *et al.*, 2007) were retrieved from BµG@Sbase and reanalysed by using Limma (Smyth, 2005). Background signals were subtracted from the foreground, and any resulting negative signals were assigned an arbitrary value of 0.5, to prevent problems arising from taking the log of a negative number. Within-array Loess normalization was performed for each array, and signals were scaled between arrays to have the same median-absolute-deviation (Smyth & Speed, 2003). Within-array replicate spot signals were averaged, and correlation of the signals for technical replicate arrays was assessed using the duplicate Correlation function (Smyth *et al.*, 2005). Differential expression was assessed by using a linear modelling approach with empirical Bayes statistics (Smyth, 2004), and the resultant *P*-values were adjusted for multiple testing using the false discovery rate controlling procedure (Benjamini & Hochberg, 1995).

#### Cellular fractionation and protein sequencing.

Bacteria were harvested from 5 ml culture (bacteria were grown as described above for RNA preparation for RNA-seq) by centrifugation in a micro-centrifuge (Hettich Mikro 22R) at 13 000 r.p.m. and 4 °C for 2 min. The supernatant was removed by aspiration, and the protein samples were resuspended in 2× final sample buffer (FSB) [0.125 M Tris/HCl (pH 6.8), 4 % SDS, 20 % glycerol, 10 % 2-mercaptoethanol, 0.05 % Bromophenol Blue], reduced with DTT and alkylated with iodoacetamide prior to separation in a 4–12 % NuPAGE Bistris gel (Invitrogen). Gels were stained with colloidal Coomassie Blue (Sigma) and bands were excised and in-gel digested with trypsin (sequencing grade; Roche). The extracted peptides were analysed with online nano liquid chromatography (LC)-MS/MS on an Ultimate 3000 Nano/Capillary LC System (Dionex) coupled to a Q-Tof Premier (Waters) or LTQ FT Ultra (ThermoElectron) mass spectrometer equipped with a nanoelectrospray source. Samples were first loaded and desalted on a PepMap C18 trap (0.3 or 0.5 mm id × 5 mm, Dionex) then separated on a C18 analytical column [Atlantis 100 µm id × 22 cm (Waters), PepMap 75 µm id × 25 cm (Dionex) or BEH 75 µm id x 10 cm (Waters)] over a 45, 60 or 90 min linear gradient of 4–32 % CH_3_CN/0.1 % FA. The Q-Tof Premier mass spectrometer was operated in the standard data-dependent acquisition (DDA) mode at a resolution of 10 000 (V mode) controlled by Masslynx 4.1. The survey scans were acquired over *m*/*z* 400–1500, and the four most abundant multiply charged ions (2^+^, 3^+^ and 4^+^) with a minimal intensity at 20 or 30 counts s^−1^ were subject to MS/MS for 3 s with dynamic exclusion for 120 s. The instrument was externally calibrated. The Raw files were processed by Proteinlynx Global Server (PLGS) 2.2.5 with and without deconvolution of MS/MS spectra by MaxEnt3. The LTQ FT Ultra mass spectrometer was operated in a similar DDA mode to the Q-Tof premier with a resolution at 100 000 at *m*/*z* 400, as described previously (Pickard *et al.*, 2010). The raw files were processed with BioWorks 3.3 (ThermoElectron). The data were subjected to a database search with Mascot Server 2.2 (Matrix Science) against an in-house-built *Campylobacter* genomic six-frame translated database using the following parameters: trypsin/P with maximum three missed cleavage sites; peptide mass tolerance at ±20 p.p.m. (FT) or 50 p.p.m. (Q-Tof Premier); MS/MS fragment mass tolerance at ±0.49 Da (FT) or 0.2 Da (Q-Tof Premier); and variable modifications, Acetyl (Protein N-term), Carbamidomethyl (C), Deamidated (NQ), Dioxidation (M), Formyl (N-term), Gln→pyro-Glu (N-term Q), Methyl (E), Oxidation (M). The Mascot result was processed with the in-house Percolator (Brosch *et al.*, 2009), and then filtered with the following rules: peptides with less than 7 amino acids or a posterior error probability >0.05 were removed. Proteins with two or more non-redundant peptides were kept. For those proteins with only one peptide identified, the peptide must be found in both datasets (Q-Tof Premier and FT) and with a PEP <0.01 and ≥10 amino acids. If the peptide was found in only one of the datasets, then the peptide must also have a Mascot score above the identification threshold. This resulted in an overall false discovery rate <0.1 %. The complete dataset from this study has been deposited in the PRIDE database (http://www.ebi.ac.uk/pride/q.do) with the accession nos 17661 and 17684.

#### Secondary structure and conservation analysis for *C. jejuni* non-coding candidates.

To identify potential unannotated ncRNA genes, large intergenic regions (>150 bp) from the complete genome sequence of *C*. *jejuni* NCTC11168 were searched against RFAMSEQ (a subset of the EMBL nucleotide database) using a combination of wu-blast filters and covariance models (Altschul *et al.*, 1990; Eddy & Durbin, 1994). Reliable matches were subsequently aligned to the reference sequence, and a consensus secondary structure was predicted using the WAR package (Torarinsson & Lindgreen, 2008); this formed a ‘seed’ alignment. Covariance models were built from the resulting alignment and secondary structure; these were searched against RFAMSEQ10 using the Rfam pipeline (Eddy & Durbin, 1994; Gardner *et al.*, 2009). New and reliable hits were added to the seed, and a new CM was built and researched. This procedure was iterated until there were no new reliable hits. The subsequent alignments were analysed using the structured RNA gene prediction methods RNAz (Washietl *et al.*, 2005) and Alifoldz (Washietl & Hofacker, 2004). These were computed using a sliding window across alignments generated from the seed sequences using clustal w (Thompson *et al.*, 1994). The window size used was 100 columns and a step-size of 10 columns for both methods. Each prediction method was run on both the forward and reverse complement strands.

## Results and Discussion

### Mapping DNA sequence reads generated by Illumina-based RNA-seq to the annotated *C. jejuni* NCTC11168 genome – global interpretations

**Fig. 1.**
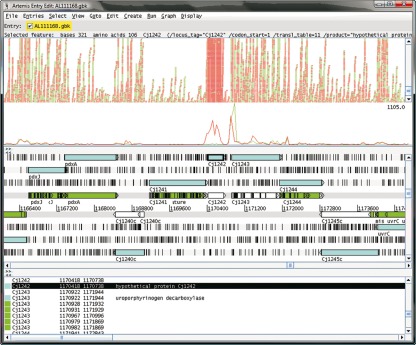
RNA-seq sequence data mapped to the *C. jejuni* NCTC11168 genome and visualized using Artemis and Bamview. The top section shows the position of reads derived from the wild-type (red) and reads from the *rpoN* : : *cat* mutant (green). The middle section shows a plot of sequence coverage, using the same colour scheme. The lower section shows a representation of the DNA strands and the six possible reading frames, and indicates the positions of annotated features. The highlighted gene, *Cj1242*, is downregulated in the *rpoN* mutant. This gene encodes the invasion antigen CiaC (Christensen *et al.*, 2009).

In order to characterize the *C. jejuni* transcriptome using RNA-seq, RNA was prepared from a pool of five independent cultures of *C. jejuni* NCTC11168 grown for 24 h to an OD_600_ 1.0 (log_10_ 10 c.f.u. ml^−1^) in BHI broth. The 16S and 23S rRNA species were depleted prior to sequencing using selective capture and magnetic separation. The resulting RNA was reverse-transcribed into cDNA which was then processed into a library of molecules that could be sequenced on the Illumina Genome Analyser. The procedure was repeated for RNA derived from a *C. jejuni rpoN* : : *cat* mutant. To assess technical variance, we sequenced each biological sample on two Illumina lanes. The sequence reads were mapped to the genome sequence of NCTC11168 and were displayed using the genome browser Artemis and Bamview (Fig. 1). The total number of reads obtained and mapped for each sample is detailed in Supplementary Table S1 (available with the online version of this paper). As previously reported for RNA-seq analysis of eukaryotic (Wilhelm *et al.*, 2008) and prokaryotic (Perkins *et al.*, 2009) RNA, the sequence coverage was not uniform across each CDS; however, our technical replicates were in close agreement (Supplementary Fig. S1), with the log_2_(RPKM+1) values for each gene and intergenic region giving Pearson correlation coefficients of 0.984 and 0.982 between replicates for the wild-type and *rpoN* : : *cat* samples, respectively (*P*<2.2×10^−16^ in both cases). Full details of the number of reads mapping to each gene and intergenic region together with log_2_(RPKM+1) values are available in Supplementary Table S2.

### Protein-coding genes

**Fig. 2.**
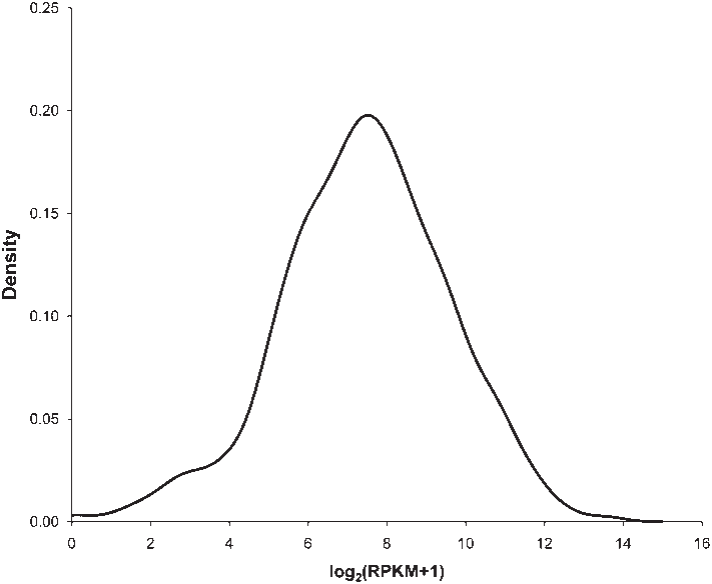
Density plot showing the distribution of log_2_(RPKM+1) values obtained for the protein-coding genes in wild-type *C. jejuni* NCTC11168.

The distribution of log_2_(RPKM+1) values for the 1624 protein-coding genes annotated in the *C. jejuni* NCTC11168 genome (Gundogdu *et al.*, 2007) is shown in Fig. 2. The distribution is approximately normal, with a mean±sd of 7.44±2.14. Only three protein-coding genes did not have any reads that mapped within their coding regions from either the wild-type or *rpoN* : : *cat* RNA-seq: *Cj0344*, *Cj0877c* and *Cj0973*. These genes are all annotated as encoding hypothetical proteins; Cj0877c is described as ‘very hypothetical’. Two more genes, annotated as encoding the hypothetical protein Cj0971 and the RepA homologue Cj1667c, had no mapped reads in the wild-type sample but did have mapped reads derived from the *rpoN* : : *cat* sample.

### Pseudogenes

**Fig. 3.**
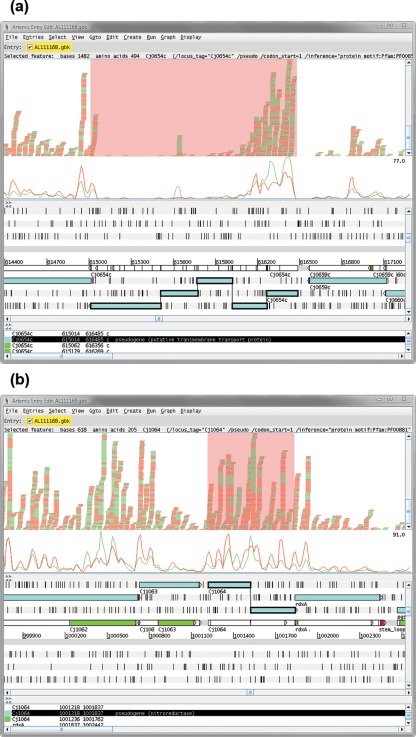
Artemis plots showing RNA-seq data obtained for pseudogenes (as for Fig. 1). (a) *Cj0654c* showed an expression pattern typical of most pseudogenes in the genome, with a high level of expression at the 5′ end of the gene that diminishes towards the 3′ end. (b) *Cj1064* showed a high level of expression across the length of the pseudogene.

The original annotation of *C. jejuni* NCTC11168 identified 20 pseudogenes (Parkhill *et al.*, 2000), and reanalysis of the genome revised this total to 19 (Gundogdu *et al.*, 2007). We were able to detect reads for all of the pseudogenes (Supplementary Table S3, available with the online version of this paper) although only four exhibited a log_2_(RPKM+1) value above the first quartile of the distribution obtained from the protein-coding genes (*Cj0501*, *Cj0565*, *Cj1064* and *Cj1528*). Closer inspection of the data indicates that most of the pseudogenes had a strong transcriptional profile at the start of the CDS with just a few isolated reads mapping to the remainder of the CDS (Fig. 3). Two of the pseudogenes (*Cj1064* and *Cj0501*) had strong transcriptional profiles throughout the entire CDS, suggesting that transcription may be maintained to express functional domains or peptides, or that the inactivation of these genes was evolutionarily recent. The sequences of our reads matched the published genome sequences for these two genes.

### tRNA

There are 43 tRNA-encoding genes annotated in the *C. jejuni* NCTC11168 genome (Gundogdu *et al.*, 2007), with 33 distinct anticodons. The log_2_(RPKM+1) values of the tRNA genes show a similar mean to the protein-coding genes (7.67; Student’s *t*-test, *P* = 0.3188), but a reduced variance (Fisher’s *F* test, *P* = 0.0040). It is a common observation that the use of alternative synonymous codons is non-random (Sharp *et al.*, 2010); in *C. jejuni* this has been attributed to the low GC-content of the genome, with a preference for AT-rich synonyms (Fuglsang, 2003). It might be expected that the expression levels of the tRNAs would reflect the codon usage bias, but this does not seem to be the case (Supplementary Tables S4 and S5, available with the online version of this paper). For example, the tRNA gene with the highest log_2_(RPKM+1) value (12.12) encodes tRNA-Arg with an anticodon CCU. However, the codon recognized by this tRNA, AGG, is used only 1340 times in the genome, whereas the synonym AGA is used 8195 times, yet its cognate tRNA with the anticodon UCU has the lowest log_2_(RPKM+1) value of all the arginine tRNAs (7.88). Leucine is the most frequently used amino acid in the *C. jejuni* proteome, yet the four tRNA-Leu genes all have average log_2_(RPKM+1) values. The levels of tRNA transcription seem to be dependent on genomic context, for example, the CCU anticodon tRNA-Arg is encoded downstream of the highly expressed *fusA* gene, and the three tRNA-Ile genes are located within rRNA operons.

### ncRNA sequences

**Fig. 4.**
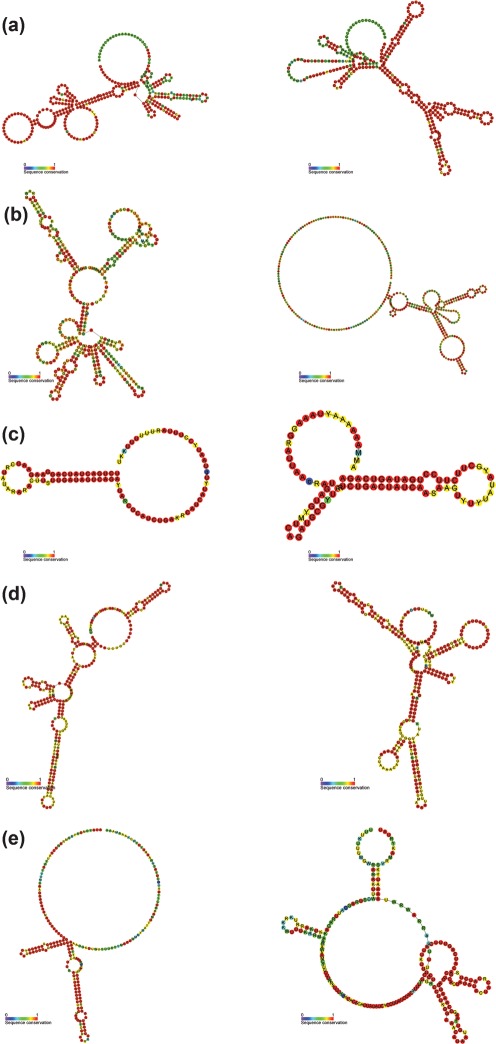
Consensus secondary structure predictions for the forward (left) and reverse (right) complement intergenic_671549–671895 (a), intergenic_722652–722740 (b), intergenic_906748–907966 (c), intergenic_1127982–1128192 (d) and intergenic_1575021–1575288 (e). The colour markup indicates the sequence conservation.

A limitation of expression microarrays is that they only provide information on specific genomic regions to which probe sequences have been designed. RNA-seq allows investigation of transcription across the whole genome, and hence enables the discovery of novel ncRNAs (Yoder-Himes *et al.*, 2009). Four ncRNAs (excluding rRNA and tRNA) are currently annotated in the *C. jejuni* NCTC11168 genome: the signal recognition particle RNA, the TPP riboswitch, the RNA component of RNaseP and 10Sa RNA (Gundogdu *et al.*, 2007). Transcripts were identified in the RNA-seq data for all of these. Additionally, we performed a screen of all large intergenic regions for potential unannotated ncRNA genes based on evolutionary conservation and structural prediction using the Rfam alignment pipeline (Gardner *et al.*, 2009), RNAz (Washietl *et al.*, 2005) and Alifoldz (Washietl & Hofacker, 2004), and identified five candidate regions which may be worth investigating further (intergenic_671549–671895, intergenic_722652–722740, intergenic_906748–907066, intergenic_1127982–1128192 and intergenic_1575021–1575288) (Table 1 and Fig. 4), although one of these (intergenic_906748–907066) did not show a significant level of expression in our data.

**Table 1.** Secondary structure and conservation analysis for *C. jejuni* ncRNA candidates ID, unique identifier containing genomic location; maxRnazP, the maximal RNAz probability on the forward or reverse strand – values greater than 0.5 were classified as putative ncRNA genes; minAlifoldZ, the lowest (best) Z-score from the RNAalifoldZ predictions on the forward or reverse strand; numSeqs, no. of homologous sequences used in the Rfam seed alignment; aveLen, average length of the sequences in the Rfam seed alignment; GC content, average G+C content for all the sequences in the Rfam seed alignment; GC enrichment, enrichment of G+C nucleotides relative to the genomic background [computed as log_2_(GC-content/0.3055)]. One homologue was identified in the *C. jejuni* genome (EMBL ACC:AL111168.1) for each ncRNA candidate.

ID no.	MaxRnazP		MinAlifoldZ		numSeqs	aveLen	aveID	GC	
	Forward	Reverse	Forward	Reverse				Content	Enrichment
Intergenic_671549–671895	0.957647	0.796566	−5.9	−5.1	8	342.9	74	0.285	−0.1001
Intergenic_722652–722740	0.995352	0.995194	−4.6	−4.4	5	89.4	89	0.295	−0.0504
Intergenic_906748–907066	0.932765	0.89473	−1.1	−6.6	5	311	89	0.232	−0.397
Intergenic_1127982–1128192	0.999921	0.559228	−8.6	−2.4	5	206.8	78	0.281	−0.1205
Intergenic_1575021–1575288	0.999869	0.999657	−7.7	−8.2	5	262.6	88	0.221	−0.4671

### RNA-seq and proteome analysis of *C. jejuni* hypothetical genes and pseudogenes

**Fig. 5.**
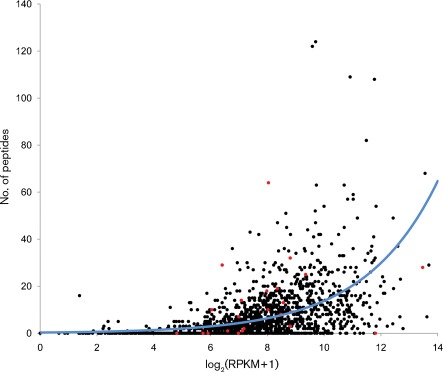
Plot of the observed number of peptides from wild-type *C. jejuni* NCTC11168 against log_2_(RPKM+1) as a measure of the mRNA expression level. The blue line indicates the predicted protein level according to the fitted log-linear regression with quasi-Poisson errors. Red points indicate mRNAs predicted to encode peptides below the 6 kDa threshold of detection.

mRNA transcript levels do not necessarily correlate with protein levels, or indeed whether the transcript is translated. We carried out a comprehensive proteomic analysis using LC-MS of peptides from whole-cell lysates of *C. jejuni* NCTC11168. We obtained good coverage with 11462 peptides mapping to 1041 predicted genes. We did not obtain any peptides that mapped to any of the pseudogenes. This might suggest that, even though we obtained transcripts for a number of the pseudogenes, they are not translated. Alternatively, any translated products may be below the level of detection or below our ~6 kDa molecular mass cut-off. Fig. 5 shows the number of peptides obtained for each protein-coding gene plotted against the log_2_(RPKM+1) value. Fitting a log-linear model with quasi-Poisson errors indicates a significant relationship between mRNA expression level and the observed peptide count (*P*<2×10^16^). For the gene *pnp* (Cj1253), a peptide sequence was obtained that included one amino acid encoded upstream of the annotated start codon. This suggests that the annotated *pnp* start codon is incorrect; the most plausible alternative is the TTG codon at position 1183537.

### RNA-seq and microarray analysis of a *C. jejuni rpoN* mutant

We generated a *C. jejuni rpoN* : : *cat* mutant and compared the growth of the mutant with the wild-type. Bacteria were cultured in MH broth with shaking in microaerobic conditions at 42 °C. The doubling time for each strain was determined for exponential phase growth: wild-type, 1.40±0.12 generations per hour; mutant 1.41±0.01 generations per hour. The difference between the growth rates was not statistically significant (Student’s *t*-test, *P*>0.05) based on viable counts. Motility assays were performed on the wild-type and *rpoN* : : *cat* mutant. Following 72 h incubation at 42 °C in microaerobic conditions, the mutant was completely non-motile (data not shown). Genes that were expressed in the *rpoN* : : *cat* mutant were identified from the RNA-seq data using DEseq (Anders & Huber, 2010). The log_2_ (fold changes) and estimated *P*-values obtained from the data are available in Supplementary Table S2. Twenty-five genes and two intergenic regions showed significantly altered expression in the *rpoN* : : *cat* mutant compared with the wild-type (*P*<0.1; see Table 2). Seventeen protein-coding genes showed significant downregulation in the *rpoN* mutant, including 12 that are annotated as encoding flagellar proteins and two others (*Cj0040* and *Cj1465*) that are co-located with flagellar gene clusters. The other three downregulated genes (*Cj0243c*, *Cj1242* and *Cj1650*) are all annotated as encoding hypothetical proteins, although Cj1242 has recently been identified as the invasion antigen CiaC, which is secreted by the *C. jejuni* flagellar type III secretion system and is potentially important in virulence (Christensen *et al.*, 2009). Cj1650 is an orthologue of the *Helicobacter pylori* protein HP1076, which has been identified as a flagellar export co-chaperone (Lam *et al.*, 2010). Two intergenic regions, located between *tpx*–*napA* (positions 731983–732128) and *luxS*–*Cj1199* (positions 1127982–1128192), were also identified as significantly downregulated. The latter region was also identified in the screen for potential ncRNA genes, suggesting the possibility of a novel ncRNA involved in flagellar regulation. Genes identified as being upregulated in the *rpoN* mutant include two gene clusters: *Cj0423–Cj0425*, which is annotated as encoding a putative integral membrane protein and two putative periplasmic proteins; and *metAB*, which encode enzymes involved in amino acid metabolism and are located close to *flgE2*. An additional membrane protein gene, *cstA*, and another amino acid metabolism gene, *cysM*, are also upregulated in the *rpoN* mutant, together with *Cj0667*, *Cj0898* and *Cj1650*, all of which are of unknown function. Interestingly, *Cj0667* is the first gene in the operon containing *rpoN*.

**Table 2.**
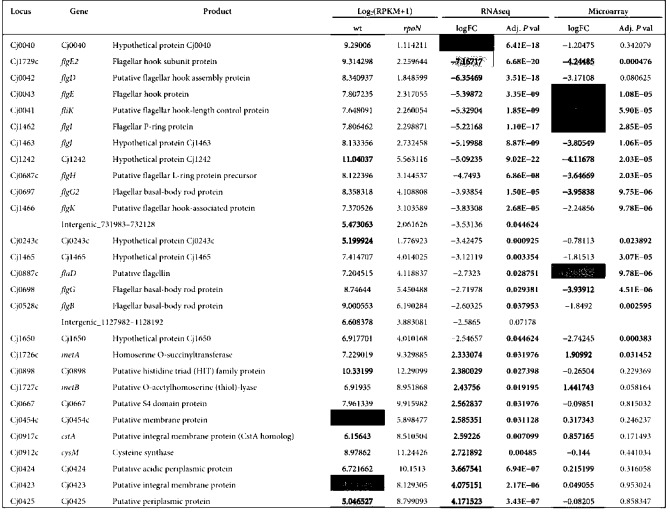
RNA-seq data for genes and intergenic regions identified as differentially expressed in the *rpoN* : : *cat* mutant relative to the wild-type The log_2_(fold change) (logFC) values for the RNA-seq and microarray, and the wild-type log_2_(RPKM+1) values are coloured by value (red >yellow >blue). RNaseq and microarray *P*-values of <0.05 are in bold type.

**Fig. 6.**
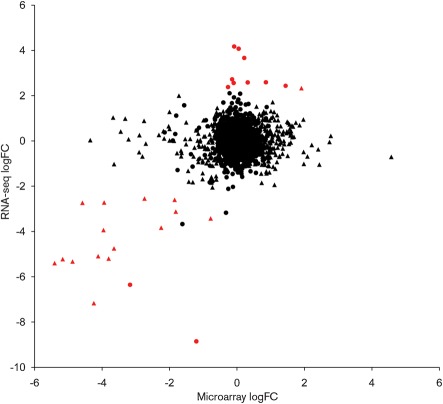
log_2_(fold change) values obtained using RNA-seq, plotted against the equivalent values from the microarray study (Kamal *et al.*, 2007). Genes showing significant differential expression in the RNA-seq data (*P*<0.05) are highlighted in red, genes showing significant differential expression in the microarray data (*P*<0.05) are shown as triangles. (Red triangles, differential expression in the RNA-seq and microarray data; red circles, differential expression in the RNA-seq data; black triangles, differential expression in the microarray data; black circles, no differential expression in either data set.)

We compared differentially expressed genes identified in the RNA-seq data with those identified in a reanalysis of data derived from an earlier microarray-based study (Kamal *et al.*, 2007). Both studies compare wild-type *C. jejuni* NCTC11168 with an *rpoN* mutant, although it should be noted that different growth conditions were employed. (Kamal *et al.* used bacteria from a 24 h plate culture, resuspended in MH broth to OD_600_ ~0.1, and grown at 37 °C for 18 h with shaking at 75 r.p.m.) All but one of the genes identified as being significantly downregulated in the *rpoN* mutant by RNA-seq were also significantly downregulated in the microarray data (Fig. 6 and Supplementary Table S2). Conversely, for the upregulated genes, only one (*metA*) was significant in both datasets. The microarray data detected considerably more significantly downregulated genes than were identified using RNA-seq. This is in part due to the additional biological replicates available for the microarray data, which add statistical power. However, most of the genes in question show no evidence of downregulation in the RNA-seq data irrespective of *P*-value, which would be unexpected were they genuinely part of the *rpoN* regulon. It is possible that the assignment of these genes as differentially expressed is an artefact of the microarray hybridization or analysis procedure. Alternatively, the differences between the two datasets could reflect the different culture conditions employed in the two studies, although we consider this unlikely. As RNA-seq becomes increasingly commonplace, it will be possible to resolve this issue by characterizing the effect of growth conditions on the *rpoN* regulon.

### Concluding remarks

In this study we have used direct high-throughput Illumina sequencing of cDNA (RNA-seq) to analyse the transcriptome of *C. jejuni* (NCTC11168). Our studies demonstrate the efficacy of high-throughput sequencing for defining mRNA expression levels and identifying differentially expressed genes and novel transcribed regions of the genome (for example, identification of potential unannotated ncRNA genes). We hope that the transcriptome and proteome datasets will be a useful resource to the *Campylobacter* research community.
